# The roles of neutrophils in non-tuberculous mycobacterial pulmonary disease

**DOI:** 10.1186/s12941-023-00562-6

**Published:** 2023-02-18

**Authors:** Meyad Alkarni, Marc Lipman, David M. Lowe

**Affiliations:** 1 Institute of Immunity and Transplantation, University College London, Pears Building, Rowland Hill Street, London, NW3 2PP UK; 2 UCL Respiratory, University College London, London, UK

**Keywords:** Neutrophils, Granulocytes, Non-tuberculous mycobacteria, Non-tuberculous mycobacterial pulmonary disease, Bronchiectasis

## Abstract

Non-tuberculous Mycobacterial Pulmonary Disease (NTM-PD) is an increasingly recognised global health issue. Studies have suggested that neutrophils may play an important role in controlling NTM infection and contribute to protective immune responses within the early phase of infection. However, these cells are also adversely associated with disease progression and exacerbation and can contribute to pathology, for example in the development of bronchiectasis. In this review, we discuss the key findings and latest evidence regarding the diverse functions of neutrophils in NTM infection. First, we focus on studies that implicate neutrophils in the early response to NTM infection and the evidence reporting neutrophils’ capability to kill NTM. Next, we present an overview of the positive and negative effects that characterise the bidirectional relationship between neutrophils and adaptive immunity. We consider the pathological role of neutrophils in driving the clinical phenotype of NTM-PD including bronchiectasis. Finally, we highlight the current promising treatments in development targeting neutrophils in airways diseases. Clearly, more insights on the roles of neutrophils in NTM-PD are needed in order to inform both preventative strategies and host-directed therapy for these important infections.

## Introduction

Non-tuberculous mycobacterial pulmonary disease (NTM-PD) is an increasingly prevalent and challenging infection syndrome that causes significant morbidity, healthcare utilization and mortality [1].

Although there are over 170 NTM species, most NTM-PD results from a small number of these environmental bacteria, which act as human opportunistic pathogens [2] but differ in their pathogenicity and response to treatment [3, 4]. Common species causing NTM-PD are *Mycobacterium avium* complex (MAC), (most often the slower-growing *M. avium, M. intracellulare* and *M*. *chimaera* species), *M. kansasii*, and *M. xenopi,* and also the rapid-grower *M. abscessus* complex (MABC) [5, 6]*.*Unlike tuberculosis, which typically affects younger people without other co-morbid illness, NTM lung disease commonly occurs in people aged fifty years or above, who may have other underlying conditions, e.g., bronchiectasis, chronic obstructive pulmonary disease (COPD) and cystic fibrosis (CF) [7–9].

The decision to treat is not straightforward: some patients appear to spontaneously clear infection and others are clinically stable without treatment over long periods of time [9]. Further, antimicrobial therapy is often prolonged, can be poorly tolerated [10] and not necessarily effective.

Studies on the host immune response to NTM have generally focused on T cells, ‘T helper-1’ cytokines and mononuclear phagocytes [11]. While these are important in protection against NTM [12], their role in either causing or preventing lung damage is less well-defined.

Generally, professional phagocytes (neutrophils, macrophages, and dendritic cells) are considered as a first-line defence against bacterial pathogens. Neutrophil polymorphonuclear granulocytes are the most prominent cell type in the bronchial lumen and are rapidly recruited to sites of infection when they sense signals from chemoattractants such as Interleukin-8 (IL-8) generated by host cells. Following migration (chemotaxis) they trap and kill the invading pathogens. Neutrophils are known to be essential for defence against extracellular bacteria [13]. However, the role of the neutrophil response in NTM-PD is still not completely understood. Recent studies have suggested that neutrophils may help control NTM infection [14, 15]; though they can also contribute to NTM-associated disease pathology, for example in the development of bronchiectasis [14, 16]. In this review we discuss the role of neutrophils in relation to non-tuberculous mycobacterial pulmonary disease (NTM-PD) and explore the apparently conflicting contributions of neutrophils in this disease.

### Neutrophils and immunity

Neutrophils’ antibacterial functions include phagocytosis (ingestion), degranulation (release of soluble antimicrobials either into the phagosome or extracellularly), and the release of nuclear material in the form of neutrophil extracellular traps (NETs) [17, 18]. Initiation of neutrophil phagocytosis is significantly enhanced by opsonisation of the bacteria whereby opsonins, for example complement components and immunoglobulins (Igs), coat the bacteria and are recognized by specific surface receptors on neutrophils causing avid binding and triggering ingestion.

Generally, once phagocytosis has been initiated by engagement of opsonic receptors, internalisation of the pathogen within phagosomes inside the neutrophil occurs in seconds [19].

Subsequently, phagosomal maturation with intracellular granule fusion takes place and gives neutrophils unique advantages over other phagocytes as the granules contain powerful bactericidal proteins. Four groups of granules are found in neutrophils: azurophil (primary), containing enzymes such as neutrophil elastase (NE) and antibacterial molecules including azurocidin and human neutrophil peptides (HNP) 1–3, specific granules (secondary), gelatinase granules, and secretory vesicles, each playing specific roles during the response to infection [20]. Some of these are discussed in more detail below.

NADPH oxidase in the wall of secondary granules initiates the oxidative burst, leading to the production of antimicrobial reactive oxygen intermediates (superoxide, hydrogen peroxide, hypochlorous acid). However, neutrophil influx may also be associated with pathology through the release of these cytotoxic contents; and if neutrophils are disrupted these processes may lead to damage of neighbouring cells and tissue injury [21, 22].

Neutrophils are also professional bacteria-responsive immune cells. Toll-like receptors (TLRs) are a type of pattern recognition receptor (PRR) that trigger the innate immune response by sensing conserved molecular patterns and allowing early immunological pathogen detection [23].

These rapid antimicrobial neutrophil functions give the acquired immune system enough time to develop pathogen-specific immunity, although, as discussed later, neutrophil behaviour can itself influence the acquired immune response.

### *The neutrophil response toNTM*

Innate phagocytic immune cells, including mononuclear phagocytes such as macrophages, rapidly eliminate mycobacteria through phagocytosis and intracellular killing, and an impairment in this process can predispose to the development of mycobacterial infection [24, 25]. Although the neutrophil response to NTM is poorly studied, previous work has proposed that granulocytes are important participants in the host defence against mycobacteria [26, 27] and these cells can kill several species of mycobacteria [28]. However they are also implicated in the pathology of mycobacterial diseases such as tuberculosis [16, 29] where they are the dominant host cell for infecting organisms in sputum, bronchoalveolar lavage (BAL) fluid, and cavity contents in patients with pulmonary TB disease [14]. Although it is clearly simplistic to translate results in *Mycobacterium tuberculosis* (Mtb)-based experiments to NTM, precise data on NTM are often lacking and in this review where necessary we have discussed the available data for Mtb. This highlights the urgent need for more research on NTM (and especially those species which cause most human disease).

Using Mtb, Jones et al. and Majeed et al. found high efficacy of mycobacterial phagocytosis by neutrophils through complement-mediated opsonization [30, 31]. However, the results of in vitro studies by Irina et al. and Lenhart-Pendergrass et al. pointed out the low capacity of neutrophils to phagocytose non-opsonized *M. smegmatis* and *M. avium* respectively [32, 33]. Collectively, these data suggest that neutrophils are capable of phagocytosing mycobacteria but this is may require opsonization by complement or immunoglobulins and could vary between species [33].

TLR-2 deficient mice with *M. avium* infection exhibited defective neutrophil function and a subsequent impairment in controlling the infection in its early stages [34, 35], implying a potentially crucial role for neutrophils in the host immune response to NTM. Conversely, it has been shown that neutrophils might contribute to the pathological dissemination of the infection rather than early clearance among genetically-susceptible mice, though this did depend on the mycobacterial species (occurring with *M. avium* but not Mtb [36]). Specifically, it has been suggested that neutrophils may carry mycobacteria to the pulmonary surface [14].

Faldt et al. reported that NTM (*M. avium and M. smegmatis*) induced a significantly higher secretion of TNFα, IL‐6, and IL‐8 from activated neutrophils than Mtb which might suggest these species evoke innate immune reactions that can lead to effective clearance of mycobacteria [37].

Table 1 summarises mouse studies suggesting a significant role for neutrophils in the early response to NTM infection, some of which are further explored in the next section.

**Table 1:** Studies suggesting a role for neutrophils in NTM infection

Study models/Reference	Mycobacteria sp	Intervention	Observations
Mouse intravenously infected 10^6^ CFU [38]	*M. avium*	Neutrophils of C57BL/6 mice infused into susceptible beige mice	Decreased the growth rate of *M. avium* compared to control beige mice
		Neutrophil depletion in C57BL/6 mice	Increased growth rate compared to control C57BL/6 mice
Mouse 10^6^ CFU or 30 mg LPS intraperitoneally 5 × 10^4^ CFU or 5 mg of LPS intratracheally [39]	*M. avium*	Gene-disrupted (CXCR2^−/−^) mice infected with *M. avium* or treated with LPS intraperitoneally/ intratracheally	Early and rapid recruitment of neutrophils with *M. avium* infection significantly impaired with CXCR2 chemokine signalling defect compared to controls
Mouse intraperitoneally infected 10^8^ CFU [27]	*M. avium*	Intravenous inoculation of mycobacteria into CD-l mice	Neutrophil phagocytosis caused degradation of the bacteria and release of enzymatic granules (lactoferrin) that increase macrophage effectiveness in eliminating mycobacteria and enhancing the further killing process
Mouse intravenously infected 10^7^ CFU [40]	*M. avium*	Administration of G-CSF into C57BL/6 black mice	Neutrophils showed anti-mycobacterial activity. Neutrophil activation inhibited growth compared with control
Mouse intravenously infected 10^6^ CFU [34]	*M. avium*	TLR2^−/−^ deficient mice infected with *M. avium*	Defect in early recruitment of neutrophils as compared to the control wild-type (WT)
Mouse intratracheally inoculated 8 × 10^7^ CFU [41]	*M. abscessus*	Wild type and cystic fibrosis mice inoculated with mycobacteria	Infection causes greater host inflammatory response based on high neutrophil number in the bronchoalveolar lavage of mice infected with rough morphotype compared to smooth morphotype in both type

*CFU* Colony Forming Unit, *G-CSF* Granulocyte-Colony Stimulating Factor, *LPS* lipopolysaccharide

### Animal models and human genetic studies implicate neutrophils in the host response to mycobacterial infection

Over 35 years ago, Brown and colleagues documented an interaction between neutrophils and mycobacteria [42]. Appelberg et al. subsequently demonstrated the major contribution of neutrophils to protect against intravenously inoculated mycobacterial infection when, using granulocyte-depleting monoclonal antibody (MAb) RB6-8C5 treatment, they noted a higher bacterial growth [38]. Petrofsky and Bermudez used a similar procedure for neutrophil depletion and also concluded that neutrophils provide some protection against *M. avium* during the early phase of infection [35]. In contrast, Saunders and Cheers did not identify a clear protective role for mouse lung neutrophils following inhalational challenge with *M. avium*, despite using similar experimental methods [43].

A study by Goncalves and Appelberg suggested that the CXC receptor 2 (CXCR2) may play a key role in neutrophil recruitment following mycobacterial infection. In comparison to control mice, the CXCR2 knockout mice had considerably fewer neutrophils in the peritoneal cavity over the course of a 15-day intraperitoneal infection with *M. avium*. However, the CXCR2 mutation had no effect on neutrophil recruitment to the lungs during an aerogenic *M. avium* infection over the course of the 60-day trial—suggesting that this may be a tissue site-related phenomenon [39].

Whole-Blood Gene Expression has been performed to investigate the host immune response to NTM-PD. A recent study included 25 patients with NTM-PD and 27 controls who were uninfected but had respiratory disease. Microarray analysis suggested that the NTM-PD population had decreased expression of 213 genes associated with T-cell signalling, including IFN-g. Chest CT lesion severity, lung dysfunction, and other markers of disease severity including high neutrophil count were associated with decreased IFN-g expression [44].

Collectively, this experimental evidence suggests that neutrophils play an important role in the host response to NTM infection though does not define what this might be, or whether it is protective or driving pathology.

### Can neutrophils kill NTM?

Several in vitro studies (summarised in Table 2) have addressed the capacity of human neutrophils to kill NTM species with the general consensus that neutrophils can eliminate—or at least restrict the growth of—clinically important NTM.

**Table 2:** In vitro studies of neutrophil ability for killing or restricting the growth of NTM

Organism	Host	Neutrophil purification	Experimental Read out	Observation	Killing / Restriction	Study reference
*M. avium*	Human (HIV)	Ficoll gradient 98–99% purity confirmed by microscopy	Radiometric assay (Bactec)	Isolated neutrophils from AIDS patients responded to exogenously supplied G-CSF by inhibiting the growth of mycobacteria	R 3–10 days	[53]
*M. avium*	Human	Ficoll sedimentation Purity NR	CFU	Half of the bacteria phagocytosed at 15 min were killed by neutrophils at 45 min, and killing was nearly complete at 120 min	K 2 h	[54]
*M. avium*	Mouse	Ficoll gradient > 97% purity confirmed by microscopy	CFU	Neutrophils from mice treated with G-CSF were able to kill *M. avium* ex vivo, compared with controls	K 4 h	[40]
*M. fortuitum*	Human	Ficoll gradient Purity NR	CFU	Killing of mycobacteria in the presence of serum, however no killing occurred in the absence of serum	K 2 h	[55]
*M. smegmatis*	Human	Percoll, > 99% purity confirmed by haematoxylin staining	CFU	Neutrophils’ antibacterial capacities demonstrated with efficient killing of mycobacteria	K Up to 6 h	[56]
*M. abscessus*	Human	Percoll, > 98% purity confirmed by microscopy	CFU	Mycobacteria activated the neutrophils’ bacterial clearance mechanisms, including ROS generation, NET formation, and phagocytosis	K 1 h	[57]

*K* Killing (reduction of CFU number), *NR* Not recorded, *R* Restriction (slower increase of CFU)

There is limited clinical evidence reporting the NTM susceptibility in neutropenic patients or those with neutrophil disorders [44]. However, neutropenia has been associated with disseminated NTM (although not pulmonary NTM) in patients with haematological malignancy [45].

A potential pathway through which neutrophils may kill mycobacteria is via human neutrophil peptides (HNP) 1, 2 and 3. These belong to a family of endogenous cationic antimicrobial and cytotoxic peptides (defensins) localised in the azurophilic granules. HNP also function as immunomodulatory molecules influencing cytokine production as well as inflammatory and immunological responses [46]. The ability of HNP-1 to kill *M. tuberculosis* has been studied in vitro by Miyakawa et al., [47], Sharma et al., [48], Kalita et al., [49], and Martineau et al., [50]. These studies have suggested that neutrophils may play a substantial role in innate resistance against TB infection through the activity of HNP and that these molecules could potentially be the basis of new therapeutic approaches.

However, another study showed that high concentrations of HNP are detected in both cystic fibrosis (CF) and non-CF bronchiectatic airways and that these inhibit PMN function via interference with phagocytosis [51, 52].

### Neutrophils directly influence the development of an acquired immune response to NTM

Neutrophils have the ability to shape adaptive immunity and bridge the innate and adaptive immune systems [58, 59].

Cytokine networks play significant roles in the cell mediated immune response to NTM infection. The T cell response against NTM is regulated by the production of IL-12 following endocytosis of mycobacteria by innate mononuclear phagocytes (eg dendritic cells (DC) and macrophages). In turn, activated CD4^+^ T cells (T-helper 1) and CD8^+^ T cells release IFNγ which enhances killing by mononuclear phagocytes and is essential for host defence against mycobacteria [60, 61]. Therefore, NTM infection which overcomes initial innate mechanisms may be controlled by efficient Th1 responses mediated by IL-12 and IFNγ [62].

Genetic mutations in the IL-12-IFNγ pathway increase susceptibility to NTM infection, for example; IFN-γR1 and IFN-γR2 deficiencies (both autosomal recessive and dominant forms), IL12β and IL12Rβ1 deficiencies, transcription factor STAT1 deficiency, RAR-related Orphan Receptor C (RORC) deficiency, interferon-stimulated gene 15 (ISG15) deficiency, interferon regulatory factor 8 (IRF8) deficiency and tyrosine kinase 2 (TYK2) deficiency [63–66].

In particular, It has been reported that defects in IL-12 and IFNγ pathways can predispose to pulmonary NTM infections [67]. Notably, interleukin-12-induced IFNγ production from T cells also activates neutrophils to phagocytose and/or kill NTM [68]. Moreover, IL-17, IL-21, and IL-22 produced by T-helper 17 CD4^+^ T cells induce neutrophil influx into inflamed disease sites which might help arrest the progression of NTM infection via direct killing [69–71].

However, neutrophil recruitment can also contribute to negative effects on the acquired immune response. A study using a mouse model of MAC infection demonstrated that when Th1 immunity is impaired, Th17 + cells provoked neutrophil recruitment that appeared to increase susceptibility to MAC infection [69]. There may be a particular negative effect of dead neutrophils. In a whole blood model with Mtb, necrotic neutrophils impaired host control of mycobacterial growth and increased immunosuppressive IL-10 as well as growth factors and chemokines [72]. This may result in further neutrophil accumulation at the site of disease: a pathological cycle that can contribute to the undesirable impact of neutrophils on host outcome [16, 73].

‘Frustrated’ neutrophils that release granule contents extracellularly could drive tissue damage and cause profound effects on T cell differentiation and proliferation [74–76]. Indeed, granule constituents or production of chemokines by neutrophils can mediate a suppressive effect directly or indirectly on T-cell responses, inactivating T-cell stimulating cytokines, eg IL-2 and IL-6, and speeding up the shedding of IL-2 and IL-6 cytokine receptors on T-cells [58, 77–79].

As an example, neutrophil elastase selectively cleaves IL-2 receptor and IL-6 receptor, and leads to the reduction of co-stimulatory molecule expression by dendritic cells, thus limiting T cell maturation and affecting the development of the Th1 response [80]. Down-regulation of T cell receptor (TCR) expression can also occur upon release of arginase and the production of reactive oxygen species (ROS) from neutrophils [58, 78].

Conversely, the production of NETs may reduce T cells’ activation threshold [81], while an abolition of Th1-specific responses has been reported when neutrophils were depleted during BCG vaccination of mice [82].

In summary, there is a bi-directional relationship between neutrophils and the acquired immune response with neutrophil behaviour potentially influencing T cell-mediated immunity either positively or negatively depending on the immune environment.

**Fig. 1:**
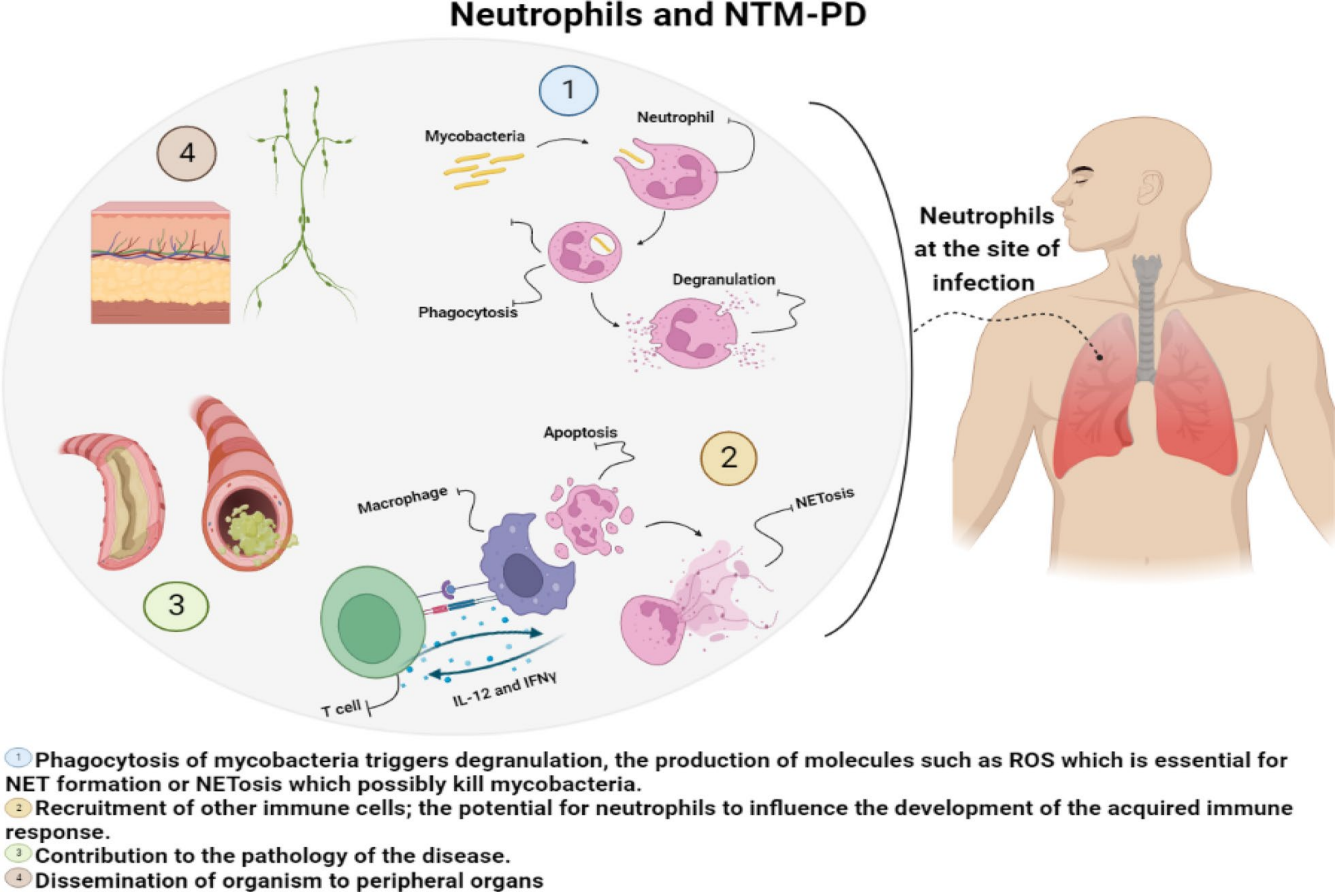
Summary of the potential roles of neutrophils in non-tuberculous mycobacterial lung disease. The figure is made with BioRender (https://app.biorender.com/). Abbreviations: ROS: Reactive oxygen species; *NET* neutrophil extracellular trap

Figure 1 summarises the various potential roles for neutrophils in NTM pulmonary disease.

### NTM, neutrophils and the humoral immune response

Mycobacteria are intracellular organisms and thus, cell-mediated immunity is considered to be the major component of host immunological defence against these bacteria. However, understanding the interaction between innate immunity, antibody-mediated immunity and cellular immunity is useful to determine strategies (both treatments and vaccines) that might combat NTM infection and disease.

Interactions of T cells, B cells and antigen presenting cells (APCs) with neutrophils allow neutrophils to modulate humoral adaptive immunity [76]. For example, activated neutrophils have a role in B-cell development through the production of B-cell activating factor (BAFF), which is an essential cytokine for B cell development, and granulocyte colony-stimulating factor (G-CSF) [76, 83]. Reciprocally, B cells can influence neutrophil activity via the production of antibodies which opsonise mycobacteria and thereby enhance neutrophil phagocytosis (discussed above).

The protective effect of the humoral immune response against mycobacterial antigens has been demonstrated in several models using Mtb. Kunnath et al. described the contribution of the humoral immune response to the control of Mtb [84] and Hamasur et al*.* showed the protective effects of mouse monoclonal IgG1 antibody to lipoarabinomannan (SMITB14) against tuberculosis when mice were infected intravenously [85]. Zimmermann et al*.* demonstrated that IgA (but not IgG) antibodies specific for different Mtb surface antigens blocked Mtb activity [86]. It is unclear whether the same applies to NTM – and this is an area which requires further investigation.

Glycopeptidolipids (GPLs) are a class of glycolipids expressed in the outer layer of several NTM species, including MAC and *M. abscessus.* The GPLs of MAC are highly antigenic and serovar-specific and are associated with MAC virulence [87, 88]. A serological diagnostic test measuring the serum IgA antibody against MAC GPLs has been developed and used clinically to diagnose MAC disease. An increase of antibody levels was recorded in patients with NTM-PD caused by MAC and not in patients with Mtb [89].

A recent study has shown the utility of serological testing in the detection of culture-positive cases of *M. abscessus* infection in CF patients [90]. The test was based on the detection of IgA against *M. abscessus* protein, recombinant PLC (rPLC), and the TLR2eF extract. This IgA ELISA was able to differentiate *M. abscessus* from *M. avium* and *M. chimaera* infections (but not from *M. intracellulare* infection) based on the recognition of MABC proteins or extracts, in contrast to the older test which is based on the detection of antibodies recognizing the GPL core antigen of *M. avium* [90]. The prevalence of NTM infection in CF patients is currently being tested in a prospective study (clinical study number ID RCB:2017-A00025-48) using both ELISAs.

Overall, studies have identified a potential role for anti-mycobacterial antibodies during the course of infection and argue for further work to help elucidate their mechanisms of action [91–94]. Specifically, antibody-mediated opsonisation of mycobacteria with subsequent enhancement of phagocytosis by neutrophils requires investigation.

### The role of neutrophils in the*** p***athology of NTM-PD

The typical pulmonary radiological patterns seen in NTM infection include bronchiectasis and cavitation, both of which are understood to be driven in large part by neutrophils, with a particular role for neutrophil elastase [95, 96].

MAC and MABC are the most prevalent species causing NTM-PD, accounting for 95% of cases [97, 98]. MABC infection, typically seen in patients with a history of pulmonary disease such as cystic fibrosis and bronchiectasis, has the highest recorded fatality rate among rapidly growing mycobacteria [3, 99–101]. MAC is less clearly associated with severe disease, but around 35–42% of positive sputum cultures for MAC represent NTM-PD [102, 103].

Why NTM are so variably pathogenic in humans is unclear*.* This is the case even with *M. avium*, whose host response is probably best understood [104].

Upon entry into the body, NTM usually settle in the lower airways and, if clinical illness develops, this presents as localised inflammation (airways disease, pneumonia, cavitation) [105].

NTM-PD is frequently seen in association with bronchiectasis, which may precede or be a consequence of the infection. In general terms, bronchiectasis can be caused by an underlying condition such as CF or other disorders of ciliary function, associated with immune deficiency (especially antibody deficiency) or occurs secondary to infection [106, 107]. It usually presents with persistent productive cough and is characterised by impairment of mucus clearance from the airways. The accumulation of mucus in the damaged airways of the lungs generates a favorable site for bacteria (including NTM) to grow, leading to further inflammation with consequent damage and dilatation of the airways, often in the right middle lobe or the lingula segment. This destruction is usually accompanied by clinical manifestations and establishes a ‘vicious circle’ due to the interaction between persistent or recurrent infection and excess inflammation [108].

Neutrophils are responsible for airway damage via the release of granule contents (human NE in particular) during degranulation [109] and are strongly implicated in the development of bronchiectasis. Granule-derived molecules have antimicrobial properties that assist in combating the infection (see above), but they can also damage host tissues (leading to bronchial dilation) [58].

Neutrophil-dominant inflammation is a central feature of bronchiectasis pathogenesis. High levels of NE in the airways are associated with exacerbations and worse lung function in both CF and non-CF bronchiectasis [110].

Some studies have reported a higher neutrophil count in the sputum of bronchiectasis patients versus healthy controls which correlates with disease progression [111–113]*.* Patients with bronchiectasis, who are at a considerably increased risk of NTM-PD [114, 115], exhibit ‘reprogramming’ of peripheral blood neutrophils during the stable state and prolonged neutrophil survival with impaired ability to kill and phagocytose bacteria, thereby perpetuating the vicious circle [116, 117]. However, this appears to improve following antibiotic treatment [116]. In addition, impairment of neutrophils’ phagocytic ability and ROS production in CF airways has been reported [118].

Any impairment of neutrophils’ ability to phagocytose and kill bacteria, including NTM, could contribute to perpetuation of the vicious circle in bronchiectasis [116].

Neutrophils can extrude the contents of their nuclei extruded to the extracellular space as neutrophil extracellular traps (NETs). NETs are made up of chromatin, histones, and various neutrophil granule proteins, including NE, cathelicidin, cathepsin G, and myeloperoxidase (MPO) [119]. NETs are used to combat pathogens in a process called NETosis, a type of cell death [120]. Cytokines such as IL8, TNF, and IFN-γ can induce NETosis in addition to bacterial components, mainly lipopolysaccharide (LPS) and lipophosphoglycan (LPG) [121].

NETs were initially identified as means of preventing bacterial dissemination by trapping and killing the bacteria [120]. However, Nakamura et al. found that MAC-induced NET formation was not involved in killing but in the production of MMPs and IL-8 that promote the progression of lung infections [122]. Furthermore, NET components such as PR3, MPO, and NE, activated and released during NETosis, are cytotoxic and have been shown to cause direct damage to the endothelium.

Moreover, research has also shown that Type I IFN-induced pulmonary NETosis can have a direct impact on TB pathogenesis in TB susceptible mice. The presence of NETs in necrotic lung lesions in patients with tuberculosis also supports a causal role for NETosis in TB pathogenesis [123, 124].

Another study has demonstrated the role of NETs in disease severity and treatment response in bronchiectasis [125], with the abundance of NET-associated proteins in patients’ sputum differing between mild and severe cases.

### Targeting neutrophils for treatment

Although they may help control NTM during early infection, neutrophils appear to have a pathogenic role in the bronchiectasis associated with established NTM-PD. Some treatments have therefore attempted to directly target neutrophils to limit further tissue damage. These have focused on neutrophil influx, neutrophil weaponry and neutrophil function (Table 3). Although no neutrophil-targeting strategies are currently licensed [126, 127], several chronic inflammatory conditions are managed—at least in part—by modifying neutrophil activity and numbers locally and systemically. These include asthma, ulcerative colitis, and rheumatoid arthritis [128].

**Table 3:** Therapies directly targeting neutrophils currently being assessed in human chronic airway diseases

Drug	Target	Indication	Mechanism of action	References
AZD9668 BAY 85–8501	NE	COPD CF Bronchiectasis	Selective NE inhibitor that elevates FEV1 and reduces inflammatory biomarkers (IL-6 and IL-8). Selective NE inhibitor that suppresses inflammation	[131, 137] [138, 139]
MK-7123	CXCR2	COPD	Reduces neutrophil chemotaxis and airway inflammation using a cytokine receptor CXCR2 antagonist (MK-7123)	[130]
AZD7986	DPP1	COPD	Blocks protease activation (reduces NE activity in the blood) via DPP1 inhibition	[140]
AZD1236	MMP	COPD	MMP-9, -12 inhibitor	[141]
GSK2269557 GSK2292767	PI3K	COPD/Asthma Asthma	Suppression of IL-8 and IL-6 levels in sputum, airway anti-inflammatory activity Inhibits neutrophil migration and degranulation	[142] [143]

^*^NE Neutrophil elastase

In the clinical setting, therapies which reduce neutrophil number are less preferable as they have been associated with compromising the patient’s immunity and increasing the risk of recurrent infections [129]. However, reduction of neutrophil migration in COPD patients seems to reduce their risk for exacerbations [126, 130].

In bronchiectasis, neutrophilic inflammation and dysfunctional killing of pathogens are considered key factors (see above). Whilst it has been proven that sputum NE is a useful marker for bronchiectasis during both stable state and exacerbations, the treatment of bronchiectasis through the inhibition of NE is still at an early stage [131]—though, this has been proposed for patients with COPD and CF using the selective NE inhibitor AZD9668 [131–133].

A recent clinical trial of brensocatib, an inhibitor of dipeptidyl peptidase 1 (DPP-1), demonstrated a relationship between the activity and quantity of neutrophil serine proteases and prognosis for patients with non-cystic bronchiectasis. A strong association was found between undetectable levels of sputum neutrophil elastase and the reduction of lung exacerbations [134].

Some therapies exist which may indirectly affect neutrophils’ function; Prezzo et al. found that intravenous immunoglobulin (IVIg) replacement therapy for antibody defects affected neutrophil activation by reducing serum IL-8 concentration, the expression of its receptor CXCR1 and the release of neutrophil elastase. This study suggested that the reduction in IL-8/CXCR1 post IVIg infusion may play a protective role in neutrophil-mediated inflammation [135]. Recently, Hitoshi et al. identified that the anti-lipoarabinomannan (anti-LAM) monoclonal IgMs, TMDU3 and LA066, significantly inhibited the phagocytosis of *Mycobacterium avium* by human neutrophils, and that mycobacterial load was reduced in the presence of neutrophils and anti-LAM IgM (albeit in the absence of other opsonins). These mannan core-directed monoclonal antibodies (mAbs) were therefore proposed as potential therapies to target aberrant or excessive neutrophil-associated immune responses [136].

Given the often poor response and considerable toxicity seen with antimicrobial therapies directed against NTM, there is an urgent need for new treatment options.

## Conclusions

In summary, available evidence suggests that neutrophils can contribute to early clearance of infection via phagocytosis and killing but may also disseminate bacilli to distant sites. Neutrophils can influence (positively or negatively) the development of acquired immune responses. In established disease, neutrophil products contribute to airway damage and are therefore appropriate targets for host-directed therapy. Currently, much of the evidence is extrapolated from research on Mtb which may not be an appropriate model for NTM, and NTM species differ between each other. Further research is required to fully characterise the diverse functions of neutrophils in NTM pulmonary disease.

## Data Availability

Not applicable.
